# Boss, Can’t You Hear Me? The Impact Mechanism of Supervisor Phone Snubbing (Phubbing) on Employee Psychological Withdrawal Behavior

**DOI:** 10.3390/healthcare11243167

**Published:** 2023-12-14

**Authors:** Siqin Yao, Ting Nie

**Affiliations:** 1 School of Business, Macau University of Science and Technology, Macau 999078, China; 3230003826@student.must.edu.mo; 2 School of Economics and Management, Taiyuan University of Science and Technology, Taiyuan 030024, China

**Keywords:** supervisor phubbing, work alienation, psychological withdrawal behavior, interpersonal sensitivity

## Abstract

With the excessive smartphone use in the workplace, supervisor phubbing has drawn broad concerns in managerial and academic fields. Though the neglect is unintentional, this behavior can generate counterproductive working behaviors. The basic assumptions of this study are that supervisor phubbing can impact employee psychological withdrawal behavior directly and indirectly via work alienation. To provide empirical evidence for the assumptions, the two-wave online survey of 302 Chinese employees without any supervisory functions was conducted on the Questionnaire Star platform. Based on the stressor-emotion model, work alienation is proved to be the psychological path in the positive relationship between supervisor phubbing and employee psychological withdrawal behavior. Different from the current studies exploring the impact mechanism of phubbing behavior on psychological withdrawal behavior between parents and children, couples, or friends, we put this mechanism into the workplace and focus on subordinate–superior relationships. In addition, the positive indirect effects are enhanced when employees have higher interpersonal sensitivity. In practice, these findings suggest that organizations should normalize the smart devices use in the workplace, and supervisors should balance their working roles with other roles. In addition, organizations should strengthen training on adjusting to negative emotions and interpersonal sensitivity control at work. Although two rounds of the time-lagged data were collected in a one-month interval, the limitations of cross-section data still exist, so the conclusions cannot establish causality. Hence, future research may conduct experimental or longitudinal research designs to make the conclusion more rigorous.

## 1. Introduction

Smartphones and the internet have become integral parts of daily life [1]. In early 2023, 92.3% of internet users, 5.16 billion people (64.4% of the global population), preferred to access the internet via smartphones; their average time spent on smartphones has exceeded five hours daily, indicating that around 30% of waking hours were spent on smartphones [2]. Particularly, the COVID-19 pandemic significantly enforces the essential role of mobile devices for telework, distance learning, collecting epidemic information, social networking online, and relieving boredom [3]. In the workplace, the average time employees spend on their smart devices doing non-work activities is roughly eight hours per workweek [4]. The excessive connectivity to mobile devices blurs the work–life boundary and impairs individual performance and wellbeing [5]. Therefore, with the explosion in smartphone use in our life [6], problematic smartphone use, such as phubbing, has emerged and drawn increasing concern about its potential adverse outcomes [7]. By merging *phone* and *snubbing*, phubbing behavior describes situations of addiction as someone (phubber) checking their mobile phones, internet, social media, and games [8], rather than paying attention to their interpersonal conversation with significant others (phubee) [9]. In this research field, a majority of attention has gone to its negative impact on the relationship between parent–child [10,11,12], partners [13,14], and friends [15,16]. In the working scenario, however, it is not uncommon that employees’ communication attempts with their supervisor are distracted or disrupted by the supervisor’s unnecessary smartphone use, known as supervisor phubbing behavior [17]. Though as an unintentional act of neglect, supervisor phubbing is enough to undermine the essential relationship with subordinates [17]. Compared with the private life domains mentioned above, however, the phubbing phenomenon in the workplace domain has been much less studied [9,17,18,19].

The present limited number of research on supervisor phubbing has been conducted from the perspectives of expectancy violations theory (EVT) [18,20,21,22], conservation of resources theory (COR) [9,21,23], social exchange theory (SET) [18,20,24], and affective events theory (AET) [1]. Among the mentioned research, supervisor phubbing is proven to have a different influential path on their subordinates in individual psychological conditions [17,24], emotions and attitudes [1,9,19,23], and working performance [9,18,25]. What is noteworthy is that phubbing can result in stressful conditions for the neglected person [23,24,26,27]. Flagged as a typical counterproductive workplace managerial behavior [19,24], supervisor phubbing is proven to negatively impact subordinates’ psychological conditions, such as producing withdrawal tendencies [6], lower employees’ organization-based self-esteem [19], engagement level, identification, and trust in their supervisor [17]. In addition, supervisor phubbing can also damage the perceived relationship quality, lead to cell phone-related conflict [28], and increase employee negative behaviors, such as employee phubbing [1] and cyberloafing [24].

Based on the integration of individual aggression and occupational stress, the stressor-emotion model of counterproductive working behavior is centered on emotions and describes the causal relationship between stressors and counterproductive working behavior via negative emotion [29]. A stressor refers to an environmental condition including a negative emotional reaction [30]. The stressor-emotion model posits that bad work events can evoke negative emotional reactions, which eventually lead to certain behaviors in reaction to these events and emotions [31]. This model concerns negative events and emotions and focuses on counterproductive working behavior as the behavioral reaction. In this research, we take supervisor phubbing behavior as the perceived stressor to employees, as the perceived stressor is fairly critical [32] and results in negative emotions, as well as we take counterproductive working behavior as the reactions [29].

Psychological withdrawal behavior (PWB) is to withdraw from work conditions psychologically [33], reflecting hateful feelings and attitudes toward the workplace or job [34,35]. Significantly, the empirical study of Wang et al. (2022a) proved the social withdrawal behavior of children as the coping mechanism of parental phubbing behavior based on attachment theory [6]. The leader–follower relationship is similar to that of parent–child [36]. In the workplace, supervisor phubbing can be considered a perceived stressor for employees, which may result in negative emotions such as work alienation—the dissociative or separate state of employees in relation to the work product or process [37]. Work alienation embodies the disconnection between people and their work [38,39]. As an essential role of task accomplishment in organizations [40], a superior–subordinate relationship, especially a poor work relationship, can be a predictor of employee alienation at work [39]. As mentioned above, supervisor phubbing may negatively impact the superior–subordinate relationship, thus resulting in work alienation. The research of Shantz et al. [37] shows that work alienation positively impacts deviance, an essential dimension of counterproductive behavior [30,41]. The stressor-emotion model illustrates a causal flow from stressor perception to behavior via emotion [29]. Work alienation reflects a negative emotional and psychological state, and supervisor phubbing can influence employees’ psychological withdrawal behavior via work alienation.

Fox and Spector [29] emphasized that the process of stress appraisal and perception is highly individual and subjective because factors such as personality and traits play a vital role in the emotional responsiveness and reaction process in the stressor-emotion model. Factors such as the centrality of non-work and other specific values [42], extraversion and neuroticism [43], and affectivity have a moderating role in forming withdrawal behaviors. Interpersonal sensitivity is a trait of someone’s awareness and sensitivity to the feelings and behaviors of others [44]. Employees with high interpersonal sensitivity are typically more sensitive to supervisor phubbing. They may feel left out and neglected, which reduces their trust in their supervisor and enhances work detachment [17]. Negative psychological perceptions can trigger more frequent withdrawal behaviors [29].

Excessive smartphone use has pervaded nearly everywhere in daily life and it is prevalent around the workplace at the same time. Despite that, the influence mechanisms of supervisor phubbing on employee counterproductive behavior are still waiting to be explored broadly and deeply. Therefore, our study aims to investigate the impact mechanisms and boundary conditions of supervisor phubbing on employee psychological withdrawal behavior. With the help of 30 Doctor of Business Administration (DBA) students, snowball sampling was conducted via a time-lagged research design. Finally, 302 valid questionnaires were returned over one-month intervals. Our research reveals the mediating effect of work alienation and the moderating effect of interpersonal sensitivity.

## 2. Literature Review and Hypotheses

### 2.1. Influence of Supervisor Phubbing Behavior on Employee Psychological Withdrawal Behavior

Supervisor phubbing, also known as boss phubbing, refers to the employees’ perception that their supervisor is distracted by the mobile device in close proximity or conversation with each other [17]. Saxena and Srivastava [24] define supervisor phubbing as the phenomenon in which a supervisor prefers snubbing the mobile phone in a meeting with the subordinate. As a counterproductive workplace managerial behavior of supervisors [19,23], supervisor phubbing can reduce the conversation intimacy and communication quality with the subordinates [45]. Based on the Conservation of Resources Theory, ill-mannered behavior, phubbing, is the source of the employee psychological resource depletion decrease [23] and the distress increase [46]. Supervisor phubbing makes them feel unappreciated, unimportant, and disrespected by a supervisor, which they view as a loss of resources (respect), thereby harming their concept and identity to themselves and their supervisor [9]. According to expectancy violation theory, supervisor phubbing may break the employees’ expectations of the supervisor’s undivided attention in a face-to-face meeting [18]. Moreover, problematic smartphone utilization and constant phone checking is proven to lead to cognitive failure in terms of forgetting others’ names, blunders that are insensible to insult the conversation partner, poor memory, and distractibility [47]. In this way, supervisor phubbing may increase cognitive failure, which would create a negative signal to employees to feel offended and ignored, thus further leading to employee counterproductive behaviors such as psychological withdrawal behavior. In addition, when interpreting this “socially unappropriated" and rude behavior from the reciprocity perspective of social exchange theory [45], supervisor phubbing expresses the signal to the employees that they are not the priority, which can reduce the sense of emotional connection and trust in their supervisor [18]. As a result, several scholars argue that subordinates view supervisor phubbing as a stressor in the workplace [23,24].

Psychological withdrawal behavior represents the neglect dimension and occurs when dissatisfied employees respond to the unsatisfactory working conditions negatively, including daydreaming, chatting excessively with co-workers, thinking of being absent, shirking in-role work, dealing with personal tasks at work, and making little effort into the job [33]. The existing research has proved that the antecedent variables of psychological withdrawal behavior include organizational support and the work–family interplay [33], qualitative job insecurity [48], instant messages demand (COR) [49], proactive personality (TAT) [50], and abusive supervision [35]. Notably, based on the attachment theory, the research on parent–children relationships proved that parent phubbing behavior can significantly predict adolescents’ social withdrawal behavior, an isolation process in which adolescents isolate themselves to stay alone [6]. Since attachment theory seeks the support role from others and has been used to investigate adult work behavior [51], we focus on the influence mechanism of phubbing behavior to social withdrawal behavior in the workplace.

Based on the integration of individual aggression and occupational stress, the stressor-emotion model of counterproductive working behavior is centered on emotions and describes the causal relationship between stressors to counterproductive working behavior via negative emotion [29]. A stressor refers to an environmental condition including a negative emotional reaction [52]. The stressor-emotion model posits that bad work events can evoke negative emotional reactions, which eventually lead to certain behaviors in reaction to these events and emotions [31]. The stressor-emotion model concerns negative events and emotions and focuses on counterproductive working behavior as the behavioral reaction. In addition, Hartanto et al. (2023) empirically proved that problematic smart device usage may result in cognitive failure. That is to say, supervisor phubbing could lead to their cognitive failure to make their subordinates feel insulted and neglected, which may incur employee work alienation [47]. In this research, we take supervisor phubbing behavior as the perceived stressor to employees, as the perceived stressor is fairly critical [32] and results in negative emotions, as well as we take counterproductive working behavior as the reactions [29]. Supervisor phubbing is also perceived as a stressor of employees, which is the gear of counterproductive working behavior. In this study, we will focus on psychological withdrawal behavior, a typical counterproductive working behavior. Based on this model, supervisor phubbing behavior will promote subordinates’ psychological withdrawal behavior as phubbing can be a stressor to them.

**Hypothesis** **1.**  *Supervisor phubbing is positively associated with employee psychological withdrawal behavior.*

### 2.2. Mediating Influence of Work Alienation

Proposed by Marx in 1932, alienation as a concept has been investigated in the subject of theology, sociology, psychiatry, and philosophy for a long time [39]. Some researchers define alienation as the external individual state or feeling of work owing to the lack of work autonomy [53]. Until the late 1960s and 1970s, this term began to receive attention in the research of organizations and management fields [54]. Among the several definitions of alienation, the most relevant to working conditions was work alienation or self-estrangement in work (i.e., engagement in work that is not intrinsically rewarding), termed by Seeman [55]. Some scholars pointed out that management practice reinforced the instrumental and unitarist viewpoint of employees [56] and led to two types of worker alienation (estrangement)—from the product (in which ownership sense and product quality control is lacking) and from the work act (in which they experience work as entirely separate from the rest of their life) [53]. In this research, we adopt the definition of Nair and Vohra [39], defining work alienation as the feelings of psychological and cognitive estrangement from employee work and context, emerging from the joint of negative and subjective psychological experiences about the work environment and context.

Work alienation derives from the individual perceptions that the work environment goes against their sense of organizational wellbeing, needs, and values [53,57,58]. The superior–subordinate relationship is proved to be a social exchange relationship continuously developing: when feeling valued, appreciated, and supported, the employees will repay them with trust and more effort in working [59]. Contrarily, if what they perceive is negative, employees will cut down their work engagement [60] and organizational commitment [61] while experiencing work alienation to remove them from work psychologically in the meantime [62]. Psychological status (such as work engagement) [63] and behaviors (such as job crafting behaviors) [64] can transform from one employee to another. The empirical evidence has proved that a supervisor’s Laissez-faire leadership can be transferred to employees’ job burnout via work alienation [65]. Therefore, counterproductive working behaviors can also transfer from the supervisor to the employees. In this research, based on the stressor-emotion model, supervisor phubbing can work as the stressor, resulting in employee psychological withdrawal behavior via work alienation as the negative emotion and psychological condition. Hence, based on the foregoing, we make the hypothesis as follows:

**Hypothesis** **2.**  *Work alienation mediates the relationship between supervisor phubbing and employee psychological withdrawal behavior.*

### 2.3. Moderating Influence of Interpersonal Sensitivity

The extent to which adverse contexts threaten individual fundamental needs varies from person to person [66] in terms of sensitivity intensity based on self-determination theory (SDT) [67]. Fox and Spector [29] pointed out that individual differences exist in counterproductive working behavior, personality, and trait, which play a critical role in the emotional responsiveness and reaction process in the stressor-emotion model.

Interpersonal sensitivity (IS) is a trait of someone’s awareness and sensitivity to the feelings and behaviors of others [44]. From the definition of Boyce and Parker [44], IS embraces two vital interpersonal components—treatment awareness and encounter reactions [68]. IS reflects the degree of interpersonal interaction sensitivity at work [69] and its level is determined via individual cognition and emotional responsiveness [70]. The prior empirical research has proved that interpersonal sensitivity can work as the boundary conditions in the impact mechanisms of compensation disadvantage on perceived compensation fairness [71], customer mistreatment on employee-displaced aggression [72], and coworkers’ envy on service performance (direct effect) and its indirect effect via relatedness needs satisfaction [70]. Moreover, employees with high interpersonal sensitivity are proven to be more sensitive to negative signals when working [69].

From the perspective of the stressor-emotion model, interpersonal sensitivity, as an employee trait, can affect the mechanism of supervisor phubbing impacts on employee psychological withdrawal behavior. Two situations are considered in which interpersonal sensitivity (IS) plays the moderator in the second stage of the mediating effect. First, employees with a higher level of interpersonal sensitivity are found to have greater awareness and response to favorable or negative treatment while the lower sensitive ones tend to be forgetful about how to be treated by others [68]. Therefore, employees with a higher level of interpersonal sensitivity are assumed to aggravate the effect of the negative emotion and psychological condition on the frequency of subsequent withdrawal behavior. Specifically, after producing the psychological status of work alienation, the relatedness needs of the subordinates with higher levels of interpersonal sensitivity can be severely affected, thereby conducting more frequent withdrawal behavior. Hence, we propose that interpersonal sensitivity may augment work alienation, the second-stage effect of the mediator, on employee withdrawal behavior.

**Hypothesis** **3.**  *Employee interpersonal sensitivity positively moderates the relationship between employee work alienation and psychological withdrawal behavior such that the relation is stronger when employee interpersonal sensitivity is high.*

**Figure 1:**
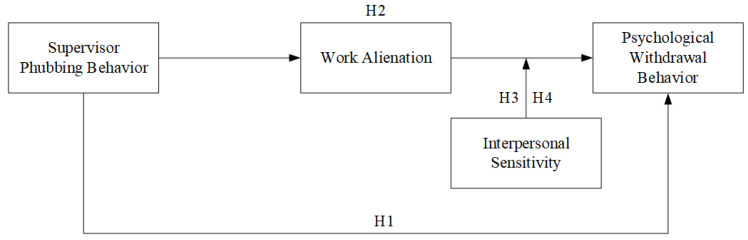
Theoretical Model.

Second, in this emotion-centered process model of counterproductive working behaviors, individuals endeavor to interpret the encountered stressors by frequently tracking and evaluating the surrounding circumstances for a variety of information and actions [30], thereby adjusting their emotional and behavioral reactions [73]. In this process, personality traits concerning emotional responsivity are also relevant to counterproductive working behavior [30]. Employees with higher levels of interpersonal sensitivity tend to feel uncomfortable and inferior with others and produce negative behaviors such as self-doubt and social avoidance [74,75], further resulting in depression, social anxiety, and other problems [72]. In this research, as analyzed above, the augmented effect of employee interpersonal sensitivity also exists in the mediating effect of work alienation between supervisor phubbing and psychological withdrawal behavior. Specifically, in the influencing process of the supervisor phubbing to psychological withdrawal behavior via work alienation, the subordinates with higher levels of interpersonal sensitivity may conduct more frequent withdrawal behavior because they feel more uncomfortable. Contrarily, employees with lower levels of interpersonal sensitivity are not sensible to others’ attitudes and behavior [44]. Hence, this group of people are supposed to produce less work alienation, and thus, conduct less frequent withdrawal behavior in face of supervisor phubbing. We propose the moderated mediating effect and theoretical model in Figure 1 as follows.

**Hypothesis** **4.**  *Employee interpersonal sensitivity positively moderates the entire indirect effect of supervisor phubbing on employee psychological withdrawal via work alienation such that the relation is stronger when employee interpersonal sensitivity is high.*

## 3. Method

### 3.1. Participants and Sampling Procedure

This study aims to examine the underlying mechanism of supervisor phubbing on employees’ psychological withdrawal and the boundary conditions of employees’ interpersonal sensitivity via an online survey of corporate employees in China. With the help of 30 Doctor of Business Administration (DBA) students, snowball sampling was used to distribute the questionnaires via “Questionnaire Star", the most widely used online data collection system in China. To reduce the effect of common method variance, the data in this study were collected in two waves with one-month intervals [76]. T1: The data on employee interpersonal sensitivity and supervisor phubbing were collected via convenience sampling. T2: The data on employee psychological withdrawal behavior, work alienation, and demographic information were collected through matching telephone numbers. A total of 560 questionnaires were distributed and 302 valid questionnaires were returned with a valid return rate of 53.0%.

The two rounds of the questionnaires’ collection lasted three months, from November 2022 to February 2023. Notably, Chinese central authorities loosened restrictions for the COVID-19 pandemic in November 2022 and many cities then experienced a surge of the pandemic affection [77]. Considering the inconvenience brought by the affection risks of omicron, during our two-time questionnaire collection, the snowball sampling method was adopted for convenience [78]. All questionnaires were distributed via “Questionnaire Star", the most widely used online data collection system in China. With the help of Doctor of Business Administration (DBA) students, the link of the online survey was sent to employees without any supervisory functions. To reduce the effect of common method variance, the data in this study were collected in two timings with one-month intervals [76]. Time 1: The data on employee interpersonal sensitivity and supervisor phubbing were collected via convenience sampling from November 2022 and lasted around one month. Time 2: After one month of the first wave of data collection, the data on employee psychological withdrawal behavior, work alienation, and demographic information were collected through matching telephone numbers. A total of 560 questionnaires were distributed and 302 valid questionnaires were returned with a valid return rate of 53.0%. In spite of telephone number matching, additional polygraph discrimination questions were applied to screen out the questionable samples that were inconsistent and 302 valid questionnaires remained, with a valid return rate of 53.0%. The item–ration ratio of this research is around 9.44 (302:32), which is much larger than the minimum ratio of 5:1 proposed by Suhr (2006) [79], so the sample size of this research is adequate.

This study was reviewed and proved by the Research Ethics Committee of the Business School at Macau University of Science and Technology (No.: MSB-20220528) in June 2022. In this study, all methods were performed in conformity with the Declaration of Helsinki. In this study, all involved subjects obtained informed consent and had our assurance of the anonymity and confidentiality of their information. At any time, they can stop answering the questionnaire, which would be considered invalid.

Among the respondents in this research, the gender ratio was relatively balanced, where males accounted for 53% (160) and females 47% (142). When it came to the respondents’ age, the age group of 18–30 and 31–40 took the majority place, with 47% and 43.4%, respectively. Most of the respondents were knowledge workers, and 84.1% (254) of them had bachelor’s or higher degrees. The industries of the respondents cover a wide range, mainly from manufacturing (31.5%), information technology (29.1%), and services (13.2%).

### 3.2. Measures

In this study, the scales were self-reported and measured with Likert 5-point scales from “complete disagreement” to “complete agreement”. SPSS 26, Amos24, and Process 3.4 were used for data analysis.

Supervisor Phubbing was measured with a 9-item scale (Cronbach’s alpha = 0.95) developed by Roberts and David [17]. An example item is “My supervisor often uses his or her cellphone when we are in meetings”.

Work Alienation was measured with an 8-item scale (Cronbach’s alpha = 0.85) proposed by Nair and Vohra [39], e.g., “I do not feel connected to the events in my workplace”.

Psychological Withdrawal Behaviorwas measured with an 8-item scale (Cronbach’s alpha = 0.81) developed by Lehman and Simpson [33], e.g., “Sometimes, I chat with co-workers about non-work topics”.

Interpersonal Sensitivity adopted the 7-item scale (Cronbach’s alpha = 0.85) developed by Boyce and Parker [44], e.g., “I worry about the effect I have on others”.

### 3.3. Techniques for Data Analysis

After collecting data from “Questionnaire Star”, which is the most widely used data collection online platform in China, the descriptive statistics and correlation analysis were conducted on SPSS 26. To examine the instrumental validity, confirmatory factor analysis (CFA) was achieved on AMOS 24. To further test the moderated mediating effect of interpersonal sensitivity, PROCESS Macro 3.4 (Model 14) was utilized for data analysis.

## 4. Results

### 4.1. Validity and Reliability

Confirmatory factor analysis (CFA) was used to test the instrument’s validity. Compared with alternative models, the overall fit of the four-factor model (supervisor phubbing, work alienation, psychological withdrawal behavior, and interpersonal sensitivity) is the best ($\chi^{2}$/*df*= 1.457, CFI = 0.967, TLI = 0.962, RMSEA = 0.039, and SRMR = 0.038). According to the most stringent guidelines, both our CFI (0.967) and TLI (0.962) are above 0.95, while both our RMSEA (0.039) and SRMR (0.038) are less than 0.06, which indicates our model has met the fit criteria and has a good approximate fit [80,81]. We also examine the average variance extracted (AVE) and composite reliability (CR) of all the constructs. As shown in Table 1, the item loadings of each variable are higher than the threshold value of 0.50; the AVE of each variable is higher than the threshold value of 0.50; and the CR of each variable is higher than the threshold value of 0.70 [82,83].

To control the effect of common method bias, we used Harman’s single-factor test as well as confirmatory factor analysis as suggested by Podsakoff et al. [76]. In Harman’s single-factor test, the variance explained by the first factor was 34.8%, which is below the threshold level of 50% [76]. Moreover, the model fit of the one-factor model in confirmatory factor analysis is far from acceptable ($\chi^{2}$/*df*= 6.227, CFI = 0.613, TLI = 0.563, RMSEA = 0.132, and SRMR = 0.092). Therefore, the common method bias in the study is not serious.

### 4.2. Descriptive Statistics and Correlation Analysis

In Table 2, the results of descriptive correlation analysis are reported. Supervisor phubbing is found to be significantly related to work alienation (r = 0.570, *p* < 0.01) and psychological withdrawal behaviors (r = 0.325, *p* < 0.01). Work alienation is also found to be significantly associated with psychological withdrawal behaviors (r = 0.378, *p* < 0.01). Therefore, the results of the preliminary correlation analysis support the hypotheses of this research.

### 4.3. Hypothesis Testing

The results of multiple regression analyses are reported in Table 3. Specifically, in the multiple regression analysis, Model 1 is to test Hypothesis 1, the main effect of supervisor phubbing on employee psychological withdrawal behavior. Models 2 and 3 are to test Hypothesis 2, the mediating role of employee work alienation. Model 4 is to test Hypothesis 3, the second stage moderating role of employee interpersonal sensitivity. Supervisor phubbing shows a positive relationship with employee psychological withdrawal behaviors in Model 1 (B = 0.325, *p* < 0.001). Thus, hypothesis 1 is supported.

Following the approach suggested by Baron and Kenny [84], Models 1 to 3 in Table 3 validate the mediating role of work alienation between supervisor phubbing and employee psychological withdrawal behavior. Supervisor phubbing is positively associated with employee psychological withdrawal behavior (B = 0.325, *p* < 0.001) and employee work alienation (B = 0.570, *p* < 0.001). When simultaneously considering both the direct effect and indirect effect on the dependent variable, work alienation is positively related to employee psychological withdrawal behavior (B = 0.286, *p* < 0.001) and the influence of supervisor phubbing on employee psychological withdrawal behavior has decreased (B = 0.162, *p* < 0.01). Therefore, the hierarchical regression analysis results show that work alienation partially mediates the relationship between supervisor phubbing and employee psychological withdrawal behavior. Thus, Hypothesis 2 is supported.

Model 4 in Table 3 validates the moderating effect of interpersonal sensitivity between work alienation and employee psychological withdrawal behavior. Work alienation (B = 0.230, *p* < 0.001), psychological withdrawal behavior (B = 0.335, *p* < 0.001), and the interaction term (B = 0.146, *p* < 0.01) are all positively associated with employee psychological withdrawal behavior. When the employee has high interpersonal sensitivity, the impact of work alienation on the employee’s psychological withdrawal behavior is stronger. Thus, Hypothesis 3 is supported.

The results of the conditional indirect effect and the moderated mediating effect are reported in Table 4 with the approach suggested by Hayes [85]. Interpersonal sensitivity moderates the relationship between supervisor phubbing, work alienation, and employee psychological withdrawal behavior (Index = 0.086; SE = 0.032; 95%CI: [0.022, 0.149]). The indirect impact of supervisor phubbing on employee psychological withdrawal behavior via work alienation is significant (Effect = 0.177, SE = 0.045; 95%CI: [0.088, 0.265]) when the employee has a high level of interpersonal sensitivity (Mean + 1SD). However, the indirect impact of supervisor phubbing on employee psychological withdrawal behavior via work alienation is not significant (Effect = 0.038, SE = 0.043; 95%CI: [−0.052, 0.119]) when the employee has a low level of interpersonal sensitivity (Mean-1SD). Thus, Hypothesis 4 is supported.

## 5. Discussions

### 5.1. Discussion of the Findings

In this study, we have tested the main effect of supervisor phubbing on employee psychological withdrawal behavior, the mediating effect of employee work alienation, the second stage of the positively moderating role of employee interpersonal sensitivity, and the positively moderated mediating role of employee interpersonal sensitivity on the indirect effect as a whole. All four hypotheses were supported by our empirical data.

Generally, our research has essential theoretical and practical contributions. First, by investigating the links between supervisor phubbing behavior and employee psychological withdrawal behavior, we shed new light on this vital issue overlooked in previous superior–subordinate research. In today’s digital era, mobile device use in working conditions is almost universal, so people must balance their work and life online and offline [17]. Supervisor phubbing has significant challenges to the relationship between employers and employees. Identifying the above reality, this research contributes to our knowledge of how supervisors’ excessive smartphone use in the workplace blurs private–professional life boundaries [7] and impairs the trust and relationship between employees and employers. Second, we clarify the underlying mediation mechanism that supervisor phubbing can influence employees’ counterproductive working [1] by raising their alienation levels. Furthermore, we propose that employees with a higher level of interpersonal sensitivity possess a poor ability to deal with supervisor phubbing, which in turn motivates them to commit psychological withdrawal behavior. Interpersonal sensitivity can worsen individuals in forming the negative consequences of supervisor phubbing and employee psychological withdrawal behavior.

### 5.2. Theoretical Contributions

The majority of the existing research on phubbing focus on the relationship between romantic partners [10,11,12], parent–child [13,14], and friends [15,16]. As for our research zooming in on the latent mechanism of phubbing between superior–subordinate relationships, this study shed new light on phubbing behavior in the workplace based on the stress-emotion model. It provides empirical evidence and enriches the limited number of studies in the working scenario. With the increasing dependency on technology and human–technology interaction [9], supervisors’ excessive use of smartphones in working conditions poses supervisor phubbing behaviors. Recent studies such as Roberts and David et al. [18], Yousaf et al. [23], and Yasin et al. [19] have acknowledged this phenomenon, with empirical evidence suggesting major results for their subordinates’ professional lives. Utilizing the stressor-emotion model, we attempted to extend prior studies by testing the impact of supervisor phubbing on employee psychological withdrawal behavior in the working scenario and the importance of work alienation as a mediator in the mechanism. In addition, interpersonal sensitivity was found to be the boundary condition of this mechanism such that a higher level of interpersonal sensitivity can promote the second stage mediating effect and the entire indirect effect. All relationships in the hypotheses were supported by empirical data from the time-lagged data in China. Based on our theoretical standpoint of the stressor-emotion model, our research contributes to the literature by illustrating an essential emotion-centered mechanism between supervisor phubbing and employee withdrawal behavior.

Digital forms of working have turned out to be the new norm in the organization, especially in the post-COVID-19 pandemic [86]. Consequently, smartphone use has also become unavoidable and essential in the workplace [23]. If employees cannot receive feedback timely or ignored by their supervisors, who instead concentrate on their mobile devices, the subordinates tend to perceive their work as less meaningful [1]. Our research findings indicate that supervisor phubbing can be a stressor to employees, thus arousing their negative emotions, thereby giving rise to their counterproductive working behavior. This mechanism can also be explained via the mirrored effect as well—employees will accept and adopt the behavioral norm more rapidly because phubbing is practiced by superiors who are supposed to be role models for their subordinates [87]. In this condition, some employees attempt to be dissociative or separated from work psychologically by negatively responding to their in-role work such as daydreaming, excessive chatting with colleagues, and shirking in-role work [33]. On the basis of the limited number of supervisor phubbing behavior literature, most of the existing studies focused its outcomes on employees’ emotions and attitudes [9,17,23], performance [18,19,21], and behaviors [1,24]. These studies generally recognized the negative impact of supervisor phubbing on their organization. In this study, we explore this situation in greater depth and concentrate on work alienation as the mediator between supervisor phubbing and employee psychological withdrawal. In line with the study of Khan [9] and Yousaf [23], we also take supervisors’ excessive use of smartphones in interaction as a stressor to their subordinates, which leads their subordinates to increase employees’ work alienation, thus makes them prone to conduct counterproductive working behaviors. These theoretical perceptions contribute to opening the black box of how supervisors’ problematic use of the smartphone impacts employee counterproductive behaviors and what managers should pay attention to regarding excessive use of smartphones and working behavior.

Another theoretical contribution of our research is our finding that interpersonal sensitivity plays an essential boundary role in worsening the mediating effect of work alienation between supervisor phubbing and employee psychological withdrawal behavior. This finding is in accordance with the existing research suggesting that individual characteristics and traits play an essential role as a vital internal cause in determining their working behaviors [72]. Specifically, different levels of sensitivity to interpersonal relationships affect the intensity of an individual’s response to unfavorable workplace experiences [69,70]. Based on the above results, our study indicates that subordinates with a higher interpersonal sensitivity are prone to feel pressure from supervisor phubbing, making it easier to become stuck into negative emotions and psychological conditions. Upon perceiving work alienation, they are more likely to engage in psychological withdrawal, a typical counterproductive working behavior.

### 5.3. Managerial Implications

Apart from the theoretical contributions, this study provides some managerial implications for employers and policymakers. First, we provide empirical evidence and directions for policymakers in the organization who try to reduce the negative effect of excessive use of smart devices in the workplace. In the workplace, when facing supervisor phubbing, the subordinates are prone to conduct psychological withdrawal behavior due to increased work alienation. What is worse, for employees with higher interpersonal sensitivity, this seems more likely the case because they are found to be more sensitive to negative signals in working conditions [69]. Second, considering employees with higher interpersonal sensitivity could be more sensitive to the negative behaviors and attitudes of others, this group of workers is more likely to be affected by supervisor phubbing behavior. In the digital era, since smart devices are hard to ban or avoid [17], effort can be devoted to training on the power of insensitivity to middle managers and their subordinates in organizations, while training employees on how to deal with their negative emotions at work. Third, our research corroborates claims that employees expect their supervisors to pay attention when they themselves are working diligently [24]. Hence, the efforts can be made to training and development to lead employees to focus more on their own in-role responsibilities and tasks. Otherwise, they may feel dissociated or detached, leading to counterproductive working behaviors. Last but not least, though interpersonal sensitivity seems to be negative in this research, what needs to be emphasized is that it is not an entirely villain trait in the workplace because a sensible manager can have better quality interactions with others because they can take notice of how others react in the interactions. Contrary to reducing the employee sensitivity level, the middle and top managers need to enhance, rather than reduce, their interpersonal level. The more sensitive they are, the higher the level of the face-to-face interaction quality with the significant others. Through the different directions of sensitive/insensitive training, interpersonal sensitivity can be controlled to the ideal level in the workplace.

## 6. Limitations and Future Study

Despite the theoretical and managerial significance, the present research still has several noteworthy limitations. First, although two rounds of the time-lagged data were collected in a one-month interval, the limitations of cross-section data still exist, so the conclusions cannot establish causality. Hence, future research may conduct experimental or longitudinal research designs to make the conclusion more rigorous [88]. In addition, because of the limited time and constrained situations of the surging affection after the relaxation of restrictions for the pandemic at the end of 2022, snowball sampling, a typical non-probability sampling, was conducted in this research. In order to obtain a more representative sample [89], the probability sampling methods should be applied in future research. Second, psychological withdrawal behavior as a typical counterproductive working behavior is a sensitive issue for employees, so the questionnaire items probably stimulate the defensive psychology of the employees. In this way, employees may hide their true feelings, opinions, or intentions and give a more positive answer, which may lead to subjects’ distorted reactions [90]. Future research may conduct multi-sourced data such as supervisor–subordinates matching methods. Third, in social interactions, the level of sensitivity of Chinese employees is found to be high to interpersonal treatment [71], which may differ from many other cultures. However, the sampling group of this research is located in China, so this may limit the external validity of our findings in this research. Fourth, we proved the partial mediating effect of work alienation in the relationship between supervisor phubbing and employee psychological withdrawal behavior. Hence, future research can make an effort to investigate other possible pathways of how supervisor phubbing impacts employee behaviors. Finally, we have to point out that we have a similar limitation to the research of Khan et al. [9]. We did not distinguish the purpose when measuring supervisor phubbing either. Work and non-work-related utilization of smartphones may have different effects during the interaction or meeting with subordinates. Future research may investigate the other reaction mechanisms of employees faced with different phubbing behaviors driven by different purposes. Additionally, the sample size of this research was 302, while the total items of our questionnaire’s main body were 32, which nearly conformed to the fundamental 10-time rule proposed by Hair et al. (2011) [91]. The item-to-ration ratio of this research is around 9.44 (302:32), which is much larger than the minimum ratio of 5:1 proposed by Suhr (2006) [92]. Therefore, it can be concluded that the sample size of this research is adequate; thus, we can preliminarily exclude the significant differences between SEM and PROCESS [93] in our results.

As the most appropriate and more conservative technique with great robustness [94], hierarchical regression analysis is a useful statistical technique for establishing and testing the significance of sets of predicted variables [95]. In addition, the objective of the overall analysis, variance analysis, required theory base, and most capabilities of PLS-SEM and linear regression are similar [94]. Considering the convenience and the limited resources and time in analysis, we tested our model using hierarchical regression and PROCESS macro, which are frequently and widely applied in many fields such as business and marketing. Even so, we still advise future research to analyze the results based on SEM software such as Mplus (https://www.statmodel.com/) or Smartpls (https://www.smartpls.com/), as Hair et al. (2011) deem PLS-SEM as the “silver bullet” [91].

## 7. Conclusions

Due to the problematic use of smartphones, it is common for both employers and employees to use personal mobile devices during interactions and meetings in the workplace. Based on the stressor-emotion model, this study investigated how supervisor phubbing induces employee psychological withdrawal behaviors. We conclude the findings as follows. First, the more supervisor phubbing is perceived, the more frequently employees conduct psychological withdrawal behavior because subordinates may view supervisor phubbing as a stressor. Since the stressor can predict counterproductive working behavior, supervisor phubbing can lead to employee psychological withdrawal. Second, in the face of supervisor phubbing, this perceived stressor may result in negative emotions and psychological conditions for employees, so employees tend to feel work alienation. In other words, supervisor phubbing may contribute to employee psychological withdrawal indirectly via work alienation. Third, employees with high interpersonal sensitivity can promote the indirect effect of work alienation on employee psychological withdrawal behavior via perceived work alienation.

## Figures and Tables

**Table 1:** Validity and reliability (n = 302).

Variables	Loadings	Cronbach’s Alpha	AVE	CR
SP	0.674–0.884	0.900	0.567	0.912
WA	0.505–0.850	0.906	0.565	0.910
PWB	0.637–0.794	0.916	0.511	0.903
IS	0.610–0.827	0.891	0.548	0.893

Note: SP: Supervisor Phubbing; WA: Work Alienation; PWB: Psychological Withdrawal Behavior; IS: Interpersonal Sensitivity.

**Table 2:** Mean, Standard Deviation, and Correlation Statistics (n = 302).

	Mean	SD	1	2	3	4
1. SP	2.36	0.616	(0.753)			
2. WA	2.11	0.691	0.570 **	(0.751)		
3. PWB	2.11	0.580	0.325 **	0.378 **	(0.715)	
4. IS	2.73	0.807	0.462 **	0.414 **	0.409 **	(0.738)

Note: ** *p* < 0.01; SD: Standard Deviation; 1 = SP: Supervisor Phubbing; 2 = WA: Work Alienation; 3 = PWB: Psychological Withdrawal Behaviors; 4 = IS: Interpersonal Sensitivity.

**Table 3:** Hierarchical regression test of mediating effect (n = 302).

	Model 1 (PWB )		Model 2 (WA)		Model 3 (PWB)		Model 4 (PWB)	
	**B**	**Se**	**B**	**Se**	**B**	**Se**	**B**	**Se**
**Independent variable**								
SP	0.325 ***	0.051	0.570 ***	0.053	0.162 **	0.061		
**Mediating variable**								
WA					0.286 ***	0.054	0.230 ***	0.047
**Moderating variable**								
IS							0.335 ***	0.041
WA × IS							0.146 **	0.048
**R^2^**	0.105		0.324		0.160		0.240	
F	35.342 ***		144.035 ***		28.576 ***		31.400 ***	

Note: *** *p* < 0.001; ** *p* < 0.01; Se: Standard Error; SP: Supervisor Phubbing; WA: Work Alienation; PWB: Psychological Withdrawal Behaviors; IS: Interpersonal Sensitivity.

**Table 4:** Moderated Mediating Effect (n = 302).

		Indirect Effect				Moderated Mediating Effect			
Path		Effect	SE	LLCI	ULCI	Index	SE	LLCI	ULCI
SP → WA → PWB	L	0.038	0.043	−0.052	0.119	0.086	0.032	0.022	0.149
	H	0.177	0.045	0.088	0.265				

Note: SE: Standard Error; SP: Supervisor Phubbing; WA: Work Alienation; PWB: Psychological Withdrawal Behavior; L: Low Level; H: High Level; LLCI: Low level of 95% confidence interval; ULCI: Upper level of 95% confidence interval.

## Data Availability

The data presented in this study are available on request from the corresponding author.

## Abbreviations

The following abbreviations are used in this manuscript:

SP	Supervisor Phubbing
WA	Work Alienation
PW	Psychological Withdrawal Behavior
IS	Interpersonal Sensitivity

## References

[1] Khan M.N., Shahzad K., Bartels J. (2022). Examining boss phubbing and employee outcomes through the lens of affective events theory. Aslib J. Inf. Manag..

[2] Kemp S. (2023). Digital 2023: Global Overview Report.

[3] Zhao J., Ye B., Yu L. (2021). Peer phubbing and Chinese college students’ smartphone addiction during COVID-19 pandemic: The mediating role of boredom proneness and the moderating role of refusal self-efficacy. Psychol. Res. Behav. Manag..

[4] Castillo L. (2023). Smartphone in the Workplace Statistics. Technical Report; Gitnux. https://blog.gitnux.com/.

[5] Yao S., Lu J., Wang H., Montgomery J.J.W., Gorny T., Ogbonnaya C. (2023). Excessive technology use in the post-pandemic context: How work connectivity behavior increases procrastination at work. Inf. Technol. People.

[6] Wang X., Qiao Y., Li W., Lei L. (2022). Parental phubbing and children’s social withdrawal and aggression: A moderated mediation model of parenting behaviors and parents’ gender. J. Interpers. Violence.

[7] Chotpitayasunondh V., Douglas K.M. (2016). How “phubbing” becomes the norm: The antecedents and consequences of snubbing via smartphone. Comput. Hum. Behav..

[8] Karadağ E., Tosuntaş Ş.B., Erzen E., Duru P., Bostan N., Şahin B.M., Çulha İ., Babadağ B. (2015). Determinants of phubbing, which is the sum of many virtual addictions: A structural equation model. J. Behav. Addict..

[9] Khan M.N., Shahzad K., Ahmad I., Bartels J. (2022). Boss, look at me: How and when supervisor’s phubbing behavior affects employees’ supervisor identification. Curr. Psychol..

[10] Xie X., Chen W., Zhu X., He D. (2019). Parents’ phubbing increases Adolescents’ Mobile phone addiction: Roles of parent-child attachment, deviant peers, and gender. Child. Youth Serv. Rev..

[11] Hu Y., Wang J., Lin Y., Zhang B. (2023). The relation of parental phubbing to academic engagement and the related mechanisms in elementary students. Learn. Individ. Differ..

[12] Jiang Y., Lin L., Hu R. (2023). Parental phubbing and academic burnout in adolescents: The role of social anxiety and self-control. Front. Psychol..

[13] Roberts J.A., David M.E. (2016). My life has become a major distraction from my cell phone: Partner phubbing and relationship satisfaction among romantic partners. Comput. Hum. Behav..

[14] Wang X., Xie X., Wang Y., Wang P., Lei L. (2017). Partner phubbing and depression among married Chinese adults: The roles of relationship satisfaction and relationship length. Personal. Individ. Differ..

[15] Kelly L., Miller-Ott A.E., Duran R.L. (2019). Phubbing friends: Understanding face threats from, and responses to, friends’ cell phone usage through the lens of politeness theory. Commun. Q..

[16] Sun J., Samp J.A. (2022). ‘Phubbing is happening to you’: Examining predictors and effects of phubbing behaviour in friendships. Behav. Inf. Technol..

[17] Roberts J.A., David M.E. (2017). Put down your phone and listen to me: How boss phubbing undermines the psychological conditions necessary for employee engagement. Comput. Hum. Behav..

[18] Roberts J.A., David M.E. (2020). Boss phubbing, trust, job satisfaction and employee performance. Personal. Individ. Differ..

[19] Yasin R.M., Bashir S., Abeele M.V., Bartels J. (2023). Supervisor phubbing phenomenon in organizations: Determinants and impacts. Int. J. Bus. Commun..

[20] Fellesson M., Salomonson N. (2020). It takes two to interact–Service orientation, negative emotions and customer phubbing in retail service work. J. Retail. Consum. Serv..

[21] Yang Z., Chen Y. The Impact of Boss Phubbing on Employees’ Job Performance: A Mediation Model with Moderation. Proceedings of the 7th International Conference on Robotics and Automation Engineering.

[22] Maftei A., Măirean C. (2023). Keep talking, I need to check my phone! Online vigilance and phubbing: The role of loneliness and moral disengagement. Ethics Behav..

[23] Yousaf S., Rasheed M.I., Kaur P., Islam N., Dhir A. (2022). The dark side of phubbing in the workplace: Investigating the role of intrinsic motivation and the use of enterprise social media (ESM) in a cross-cultural setting. J. Bus. Res..

[24] Saxena A., Srivastava S. (2023). Is Cyberloafing an Outcome of Supervisor Phubbing: Examining the Roles of Workplace Ostracism and Psychological Detachment. Int. J. Bus. Commun..

[25] Karimikia H., Singh H., Joseph D. (2020). Negative outcomes of ICT use at work: Meta-analytic evidence and the role of job autonomy. Internet Res..

[26] Wang X., Gao L., Yang J., Zhao F., Wang P. (2020). Parental phubbing and adolescents’ depressive symptoms: Self-esteem and perceived social support as moderators. J. Youth Adolesc..

[27] Wang H., Zhou L., Geng J., Lei L. (2022). Sex differences of parental phubbing on online hostility among adolescents: A moderated mediation model. Aggress. Behav..

[28] Halpern D., Katz J.E. (2017). Texting’s consequences for romantic relationships: A cross-lagged analysis highlights its risks. Comput. Hum. Behav..

[29] Fox S., Spector P.E. (2005). Counterproductive Work Behavior: Investigations of Actors and Targets.

[30] Spector P.E., Fox S., Penney L.M., Bruursema K., Goh A., Kessler S. (2006). The dimensionality of counterproductivity: Are all counterproductive behaviors created equal?. J. Vocat. Behav..

[31] Eissa G., Lester S.W., Gupta R. (2020). Interpersonal deviance and abusive supervision: The mediating role of supervisor negative emotions and the moderating role of subordinate organizational citizenship behavior. J. Bus. Ethics.

[32] Perrewé P.L., Zellars K.L. (1999). An examination of attributions and emotions in the transactional approach to the organizational stress process. J. Organ. Behav..

[33] Lehman W.E., Simpson D.D. (1992). Employee substance use and on-the-job behaviors. J. Appl. Psychol..

[34] Sagie A., Birati A., Tziner A. (2002). Assessing the costs of behavioral and psychological withdrawal: A new model and an empirical illustration. Appl. Psychol..

[35] Huang L.C., Lin C.C., Lu S.C. (2020). The relationship between abusive supervision and employee’s reaction: The job demands-resources model perspective. Pers. Rev..

[36] Keller T., Cacioppe R. (2001). Leader-follower attachments: Understanding parental images at work. Leadersh. Organ. Dev. J..

[37] Shantz A., Alfes K., Bailey C., Soane E. (2015). Drivers and outcomes of work alienation: Reviving a concept. J. Manag. Inq..

[38] Mottaz C.J. (1981). Some determinants of work alienation. Sociol. Q..

[39] Nair N., Vohra N. (2009). Developing a new measure of work alienation. J. Workplace Rights.

[40] Lee J., Jablin F.M. (1995). Maintenance communication in superior-subordinate work relationships. Hum. Commun. Res..

[41] Wu J., Lebreton J.M. (2011). Reconsidering the dispositional basis of counterproductive work behavior: The role of aberrant personality. Pers. Psychol..

[42] Mobley W.H., Griffeth R.W., Hand H.H., Meglino B.M. (1979). Review and conceptual analysis of the employee turnover process. Psychol. Bull..

[43] Ilies R., Judge T.A. (2002). Understanding the dynamic relationships among personality, mood, and job satisfaction: A field experience sampling study. Organ. Behav. Hum. Decis. Process..

[44] Boyce P., Parker G. (1989). Development of a scale to measure interpersonal sensitivity. Aust. N. Z. J. Psychiatry.

[45] Abeele M.V., Hendrickson A.T., Pollmann M.M., Ling R. (2019). Phubbing behavior in conversations and its relation to perceived conversation intimacy and distraction: An exploratory observation study. Comput. Hum. Behav..

[46] Tandon A., Dhir A., Talwar S., Kaur P., Mäntymäki M. (2022). Social media induced fear of missing out (FoMO) and phubbing: Behavioural, relational and psychological outcomes. Technol. Forecast. Soc. Chang..

[47] Hartanto A., Lee K.Y., Chua Y.J., Quek F.Y., Majeed N.M. (2023). Smartphone use and daily cognitive failures: A critical examination using a daily diary approach with objective smartphone measures. Br. J. Psychol..

[48] Vander Elst T., Richter A., Sverke M., Näswall K., De Cuyper N., De Witte H. (2014). Threat of losing valued job features: The role of perceived control in mediating the effect of qualitative job insecurity on job strain and psychological withdrawal. Work Stress.

[49] Jiang X., Du J., Yang T., Liu Y. (2021). How do instant messages reduce psychological withdrawal behaviors?—Mediation of engagement and moderation of self-control. Int. J. Environ. Res. Public Health.

[50] Song C., Lee C.H. (2020). The effect of service workers’ proactive personality on their psychological withdrawal behaviors: A moderating effect of servant leadership. Leadersh. Organ. Dev. J..

[51] Wu C.H., Parker S.K. (2017). The role of leader support in facilitating proactive work behavior: A perspective from attachment theory. J. Manag..

[52] Spector P.E., Jex S.M. (1998). Development of four self-report measures of job stressors and strain: Interpersonal conflict at work scale, organizational constraints scale, quantitative workload inventory, and physical symptoms inventory. J. Occup. Health Psychol..

[53] Sarros J.C., Tanewski G.A., Winter R.P., Santora J.C., Densten I.L. (2002). Work alienation and organizational leadership. Br. J. Manag..

[54] Seybolt J.W., Gruenfeld L. (1976). The discriminant validity of work alienation and work satisfaction measures. J. Occup. Psychol..

[55] Seeman M. (1967). On the personal consequences of alienation in work. Am. Sociol. Rev..

[56] Kanungo R.N. (1992). Alienation and empowerment: Some ethical imperatives in business. J. Bus. Ethics.

[57] Wegner E.L. (1975). The concept of alienation: A critique and some suggestions for a context specific approach. Pac. Sociol. Rev..

[58] Kanungo R.N. (1979). The concepts of alienation and involvement revisited. Psychol. Bull..

[59] Guo L. (2020). The effect of workplace loneliness on silence behavior. Psychology.

[60] Kulik C.T., Perera S., Cregan C. (2016). Engage me: The mature-age worker and stereotype threat. Acad. Manag. J..

[61] Dong R., Yu W., Ni S., Hu Q. (2022). Ageism and employee silence: The serial mediating roles of work alienation and organizational commitment. Ethics Behav..

[62] Banai M., Reisel W.D., Probst T.M. (2004). A managerial and personal control model: Predictions of work alienation and organizational commitment in Hungary. J. Int. Manag..

[63] Bakker A.B., Emmerik H.v., Euwema M.C. (2006). Crossover of burnout and engagement in work teams. Work Occup..

[64] Peeters M.C., Arts R., Demerouti E. (2016). The crossover of job crafting between coworkers and its relationship with adaptivity. Eur. J. Work Organ. Psychol..

[65] Usman M., Ali M., Yousaf Z., Anwar F., Waqas M., Khan M.A.S. (2020). The relationship between laissez-faire leadership and burnout: Mediation through work alienation and the moderating role of political skill. Can. J. Adm. Sci. Can. Sciences L’Adm..

[66] Deci L., Ryan M. (2000). The “what” and “why” of goal pursuits: Human needs and the self-determination of behavior. Psychol. Inq..

[67] Exline J.J., Lobel M. (1999). The perils of outperformance: Sensitivity about being the target of a threatening upward comparison. Psychol. Bull..

[68] Wu L.Z., Tse E.C.Y., Fu P., Kwan H.K., Liu J. (2013). The impact of servant leadership on hotel employees’ “servant behavior”. Cornell Hosp. Q..

[69] Bunk J.A., Magley V.J. (2011). Sensitivity to interpersonal treatment in the workplace: Scale development and initial validation. J. Occup. Organ. Psychol..

[70] Liu X., Wu L.Z., Ye Y., Liu L., Cheng X.M. (2023). Green-eyed coworkers in service organizations: The impact of being envied by coworkers on employee service outcomes. Asia Pac. J. Manag..

[71] Chen C.C., Choi J., Chi S.C. (2002). Making justice sense of local-expatriate compensation disparity Mitigation by local referents, ideological explanations, and interpersonal sensitivity in China-foreign joint ventures. Acad. Manag. J..

[72] Liu F., Chen G., Liu Y. (2020). The impact of customer mistreatment on employee displaced aggression: The moderating effect of interpersonal sensitivity and moral identity. Front. Psychol..

[73] Matta F.K., Erol-Korkmaz H.T., Johnson R.E., Biçaksiz P. (2014). Significant work events and counterproductive work behavior: The role of fairness, emotions, and emotion regulation. J. Organ. Behav..

[74] Davidson J., Zisook S., Giller E., Helms M. (1989). Symptoms of interpersonal sensitivity in depression. Compr. Psychiatry.

[75] Otani K., Suzuki A., Ishii G., Matsumoto Y., Kamata M. (2008). Relationship of interpersonal sensitivity with dimensions of the Temperament and Character Inventory in healthy subjects. Compr. Psychiatry.

[76] Podsakoff P.M., MacKenzie S.B., Lee J.Y., Podsakoff N.P. (2003). Common method biases in behavioral research: A critical review of the literature and recommended remedies. J. Appl. Psychol..

[77] Li L. (2022). Cities Brace for Surge in COVID Infections.

[78] Rahman M.M., Khan S.J., Sakib M.S., Chakma S., Procheta N.F., Mamun Z.A., Arony A., Rahman F., Rahman M.M. (2021). Assessing the psychological condition among general people of Bangladesh during COVID-19 pandemic. J. Hum. Behav. Soc. Environ..

[79] Rahman M.M. (2023). Sample Size Determination for Survey Research and Non-Probability Sampling Techniques: A Review and Set of Recommendations. J. Entrep. Bus. Econ..

[80] Floyd F.J., Widaman K.F. (1995). Factor analysis in the development and refinement of clinical assessment instruments. Psychol. Assess..

[81] Hu L.t., Bentler P.M. (1998). Fit indices in covariance structure modeling: Sensitivity to underparameterized model misspecification. Psychol. Methods.

[82] Fornell C., Larcker D. (1981). Evaluating structural equation models with unobservable variables and measurement error. J. Mark. Res..

[83] Hair J., Hollingsworth C.L., Randolph A.B., Chong A.Y.L. (2017). An updated and expanded assessment of PLS-SEM in information systems research. Ind. Manag. Data Syst..

[84] Baron R.M., Kenny D.A. (1986). The moderator–mediator variable distinction in social psychological research: Conceptual, strategic, and statistical considerations. J. Personal. Soc. Psychol..

[85] Hayes A.F. (2017). Introduction to Mediation, Moderation, and Conditional Process Analysis: A Regression-Based Approach.

[86] Adisa T.A., Ogbonnaya C., Adekoya O.D. (2023). Remote working and employee engagement: A qualitative study of British workers during the pandemic. Inf. Technol. People.

[87] Krasnova H., Abramova O., Notter I., Baumann A. Why phubbing is toxic for your relationship: Understanding the role of smartphone jealousy among “Generation Y” users. Proceedings of the ECIS 2016 Conference.

[88] Lu J., Guo Z., Usman M., Qu J., Fareed Z. (2023). Conquering precarious work through inclusive leadership: Important roles of structural empowerment and leader political skill. Hum. Relations.

[89] Rahman M.M., Tabash M.I., Salamzadeh A., Abduli S., Rahaman M.S. (2022). Sampling techniques (probability) for quantitative social science researchers: A conceptual guidelines with examples. SEEU Rev..

[90] Nie T., Qiu T. (2019). An Empirical Study on the Mechanism of Cyberloafing: Based on the Stressor-Emotion Model. Chin. J. Manag..

[91] Hair J.F., Ringle C.M., Sarstedt M. (2011). PLS-SEM: Indeed a silver bullet. J. Mark. Theory Pract..

[92] Suhr D. (2006). Exploratory or Confirmatory Factor Analysis. Proceedings of the SAS Users Group International Conference.

[93] Hayes A.F., Montoya A.K., Rockwood N.J. (2017). The analysis of mechanisms and their contingencies: PROCESS versus structural equation modeling. Australas. Mark. J..

[94] Gefen D., Straub D., Boudreau M.C. (2000). Structural equation modeling and regression: Guidelines for research practice. Commun. Assoc. Inf. Syst..

[95] Tisak J. (1994). Determination of the regression coefficients and their associated standard errors in hierarchical regression analysis. Multivar. Behav. Res..

